# Global trends in inappropriate use of antibiotics, 2000–2021: scoping review and prevalence estimates

**DOI:** 10.1136/bmjph-2024-002411

**Published:** 2025-05-27

**Authors:** Ranya Mulchandani, Katie Tiseo, Arindam Nandi, Eili Klein, Sumanth Gandra, Ramanan Laxminarayan, Thomas Van Boeckel

**Affiliations:** 1 Health Geography and Policy Group, ETH Zurich, Zurich, Switzerland; 2 Population Council, New York, New York, USA; 3 One Health Trust, Washington, District of Columbia, USA; 4 Johns Hopkins University, Baltimore, Maryland, USA; 5 Division of Infectious Diseases, Department of Medicine, Washington University, Saint Louis, Missouri, USA; 6 One Health Trust India, Bengaluru, India; 7 Princeton University, Princeton, New Jersey, USA; 8 One Health Institute, University of Zurich, Zurich, Switzerland; 9 Spatial Ecology and Epidemiology Lab, Université Libre de Bruxelles, Brussels, Belgium

**Keywords:** Public Health, Population Surveillance, Scoping Review, Prevalence, Epidemiologic Methods

## Abstract

**Introduction:**

Inappropriate antibiotic use is a major driver of antimicrobial resistance. However, the scope of literature and its prevalence across world regions remain largely unknown, as do the most common indicators and study designs used. In this study, we summarised the current literature on inappropriate use of antibiotics by world regions. We also provided the first global estimates of the overall amount of antibiotics that are potentially used inappropriately each year.

**Methods:**

We considered both patient and provider-mediated inappropriate antibiotic use. We reviewed 412 studies published between 2000 and 2021 and used beta regression and marginal contrasts to compare prevalence of inappropriate use by study design, indicator, world region, and national income level. Country-level sales of antibiotics from 2022 were combined with inappropriate antibiotic use estimates derived from two study designs (clinical audits and patient interviews) and one indicator (lack of indication) to estimate the amount of antibiotics inappropriately used globally.

**Results:**

Clinical audits (50.1%, 208/412) and ‘non-prescription’ use (37.1%, 153/412) were the most common study design and indicator, respectively, used to estimate inappropriate antibiotic use. Inappropriate antibiotic use prevalence was ~6% higher in low-income and middle-income than in high-income countries. However, this difference disappeared after accounting for a proxy of access to care: physicians per capita. Globally, based on clinical audits, patient interviews and lack of indication, the estimated proportion of inappropriate antibiotic use was 29.5%, 36.5% and 30.8%, respectively, with an average of ~30% (~13 000 000 kg) the equivalent of the annual antibiotic consumption in China.

**Conclusions:**

Inappropriate antibiotic use is highly prevalent across all countries regardless of national income level, with a third of global antibiotic consumption potentially due to unnecessary prescription (‘lack of indication’). Antibiotic stewardship efforts and defining internationally standardised indicators are needed to track progress in reducing the occurrence of inappropriate antibiotic use where necessary, as well as identifying gaps in access to care.

**WHAT IS ALREADY KNOWN ON THIS TOPIC**

- In 2021, Sweileh reviewed bibliometric data on the published literature on ‘irrational’ use of antimicrobials worldwide. However, the analysis did not attempt to quantify the prevalence of inappropriate antibiotic use globally, or by world region, study design and indicator. Two further studies (Kardas *et al* and Morgan *et al*) estimated global prevalence of inappropriate use of antibiotics, but these focused on specific subsets of inappropriate use: the first study estimated the prevalence of ‘misuse’ of antibiotics in the community only, while the second focused solely on the prevalence of ‘non-prescription’ antimicrobial use.

**WHAT THIS STUDY ADDS**

- This study summarises the current evidence available on study designs and indicators used to quantify the prevalence of inappropriate use of antibiotics worldwide since 2000. Prevalence of inappropriate antibiotic use was highly heterogeneous depending on study design (eg, patient interviews, clinical audits) and indicator used (eg, incorrect dosage, no indication). Prevalence was found to be higher in low-income and middle-income countries. However, these differences disappeared after accounting for the number of physicians per capita to reflect access to healthcare/antibiotic prescriptions. We estimated the global quantity of potentially inappropriately used antibiotics under three scenarios based on the prevalence of inappropriate use in clinical audits (29.5%), patient interviews (36.5%) and studies focusing on lack of indication (30.8%).

**HOW THIS STUDY MIGHT AFFECT RESEARCH, PRACTICE OR POLICY**

- Our findings indicate that standardised studies to quantify inappropriate use of antibiotics are urgently needed in all parts of the world because current estimates of prevalence of inappropriate use of antibiotics are highly heterogeneous depending on world regions, study designs and indicators considered. Working within these limitations, our attempts at estimating the prevalence of inappropriate antibiotic use suggest that a third of antibiotics consumed globally may be associated with unnecessary prescriptions by healthcare workers. The prevalence of inappropriate use could reach as much as 37% when estimated from inappropriate dosage, inappropriate duration or inappropriate choice of antibiotics, indicating the need for considerable stewardship efforts. In addition, inappropriate use in low-income and middle-income countries is likely driven by limited access to healthcare providers, and this should be accounted for in future efforts that systematically quantify inappropriate use of antibiotics. Proposed global targets on reducing inappropriate use will require standardised indicators and estimation approaches to supplement this initial attempt at characterising inappropriate antibiotic use globally.

## Introduction

 Inappropriate use of antibiotics harms patients and contributes to the rise of antimicrobial resistance (AMR).^1^,^3^ In children, early antibiotic exposure could lead to adverse long-term health outcomes, such as asthma, obesity and neurodevelopmental disorders.^4^ Exposure to antibiotics is associated with potential side effects, as well as risks of reduced efficacy of future use of antibiotics due to the emergence of resistant bacteria.^5^ The societal effects of AMR are also significant: it is associated with increased treatment failure, severity and complications of infections, comparatively longer hospital stays and high healthcare costs.^6 7^ In contrast to recent efforts to quantify the global burden of AMR^8^ and global antimicrobial use (AMU),^9^ limited efforts have been made to document inappropriate use of antibiotics globally, or its relative prevalence between world regions, national income level and clinical specialties.

The absence of a global assessment on inappropriate use of antibiotics likely stems from its multifaceted nature and lack of a consensus definition. The WHO defines ‘rational use of medicines’ as *“patients [that] receive medications appropriate to their clinical needs, in doses that meet their own individual requirements, for an adequate period of time, and at the lowest cost to them and their community”*.^10^ However, even though this definition extends to antimicrobials, what is determined as rational use varies with clinical context, making it challenging to apply the same guidelines across all infectious conditions. As such, previous analyses have thus far used heterogeneous measures of inappropriate antibiotic use and/or have focused on subsets of inappropriate use, such as non-compliance with clinical guidelines,^11^ inappropriate fixed-dose combinations,^12^ or concentrated in certain regions of the world such as the Middle East^13^ or low-income and middle-income countries (LMICs).^14^ Inappropriate use of antibiotics can be influenced locally by multiple factors, including lack of regulations or enforcement thereof and limited access to prescribers, particularly in LMICs. Furthermore, inappropriate use of antibiotics has been shown to differ by gender,^13 15^ age^16^ and education or income levels,^1315^,^17^ which vary widely by regions.

Inappropriate use of antibiotics has been quantified across studies using a diverse range of indicators and study designs.^11 18^ However, to our knowledge, the study designs most commonly used to quantify inappropriate antibiotic use have not yet been summarised, nor have patient and provider-mediated inappropriate use been compared. This currently limits our ability to interpret published estimates on inappropriate use of antibiotics and to estimate the global prevalence of inappropriate use of antibiotics. In 2021, one study used bibliometric metadata to investigate trends in what they termed ‘irrational’ use of antimicrobials globally.^19^ A second review in 2021 attempted to understand the factors associated with providers, patients, health system and pharmaceutical industries to explain antibiotic ‘overuse’.^20^ In 2024, a review of knowledge, attitudes and practices (KAPs) attempted to identify clinicians’ motivations for prescribing antibiotics.^21^ However, crucially, none of these works documented the variety of indicators and study designs used to estimate inappropriate use of antibiotics, nor did they attempt to quantify its global prevalence, thereby limiting their potential for guiding quantifiable stewardship efforts.

In this study, we describe the current evidence (or lack thereof) on inappropriate antibiotic use and identify the most common categories of indicators and study designs used to estimate it. Second, we compared the prevalence of inappropriate antibiotic use by study design, indicator, world region and national income level. Third, we quantified the amount of antibiotics potentially used inappropriately worldwide by combining global estimates of antibiotic use per country in 2022^9^ with prevalence of inappropriate antibiotic use estimated from two study designs, clinical audits (provider-mediated use) and patient interviews (patient use), as well as one indicator, the lack of indication (provider-mediated use).

## Methods

### Data collection

#### Search strategy and selection criteria

**Figure 1:**
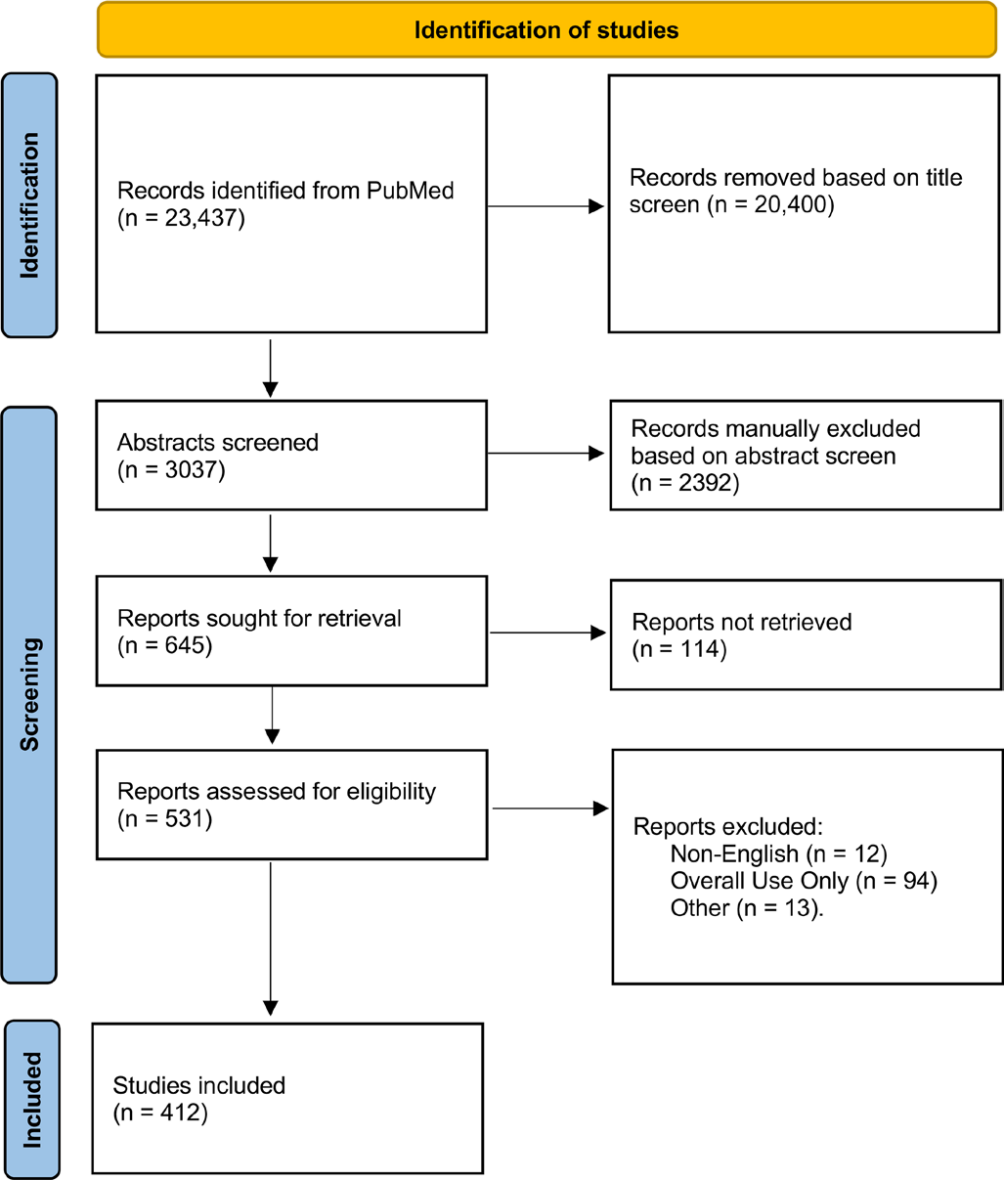
PRISMA flow diagram of study inclusion and exclusion. PRISMA, Preferred Reporting Items for Systematic Reviews and Meta-Analyses.

We conducted a scoping literature review in PubMed on 1 December 2022, for peer-reviewed studies published in English that reported inappropriate use of antibiotics between 1 January 2000 and 31 December 2021. We attempted to build a broad and inclusive picture of a multifaceted topic that encompasses multiple definitions of inappropriate use of antibiotics. Therefore, instead of collating evidence on a narrowly defined research question and search terms (ie, systematic review), we conducted a scoping review to reflect the diversity of study designs and indicators used to study inappropriate use. For this study, we considered both patient-mediated and provider-mediated use of antibiotics that was not considered ‘appropriate’. Our search term followed a format of “misuse” (misuse, overuse, abuse, inappropriate*, irrational, unnecessary, excessive, self-prescri*, self-medicat*, non-prescription, community pharmacy, misprescri*, non-compliance, non-adherence, incorrect, incomplete, improper) AND “antimicrobial” (antibiotic*, antimicrobial*). Studies of inappropriate use in animals or the environment were not included, nor were papers where only overall use was described (ie, no breakdown of appropriate vs inappropriate use). Text screening followed a stepwise protocol, with exclusion and inclusion criteria detailed in figure 1 and reported in accordance with the Preferred Reporting Items for Systematic Reviews and Meta-Analyses Extension for Scoping Reviews (online supplemental file 1). All data extracted in this review are available for download (online supplemental file 2).

#### Data extraction

Data were extracted from the studies using a standardised data extraction template, which included publication year; country; study design (grouped into: clinical audit, patient interview, provider interview or behavioural studies); sample size (the number of individuals receiving antibiotics, except for community-based surveys, where the number of individuals responding to the survey was used as a proxy); population under study; drug class; drug; estimated inappropriate use of antibiotics (%); CI; indicator used to categorise inappropriate use (see next section); key term(s) used to describe the type of inappropriate usage; and whether the inappropriate use of antibiotics was estimated following an intervention (eg, proportion of antibiotics inappropriately used before/after an introduction of a new antibiotic stewardship intervention in a hospital). We did not analyse inappropriate use by antibiotic class due to the limited number of studies (17.0%, 176/1033) reporting this information.

### Classification of inappropriate use: study designs and indicators

Studies of inappropriate use were classified into four categories based on study design:

#### Clinical audit/database analysis

These studies were typically conducted in a hospital or medical doctor’s practice. Clinical information on antibiotics prescribed was obtained from clinical databases. These data were reviewed and defined as whether an antibiotic prescription did or did not match the clinical prescribing guidelines recommended at the study location for the clinical indication. This type of study measured provider-mediated use of antibiotics.

#### Patient interviews

These studies measured patient use of antibiotics. Patients were asked questions, either in-person or remotely, in either a clinical setting (eg, pharmacy) or in the community. Sampled populations included either those with symptoms where an antibiotic may be required, where individuals have received antibiotics, or general populations inquiring about routine antibiotic use. Questions varied by sampling population. For instance, they may concern patients’ attitudes, for example, *“Did you not receive an antibiotic when you believed you should have received one?”,* or practices for example, *“Will you complete your full antibiotic prescription even on succession of symptoms?”*, or *“Have you taken an antibiotic without a prescription in the last 6 months?”*. All antibiotics taken in the absence of a prescription (‘non-prescription’) were classified as inappropriate use.

#### Provider interview

These studies measured provider-mediated use of antibiotics and included surveys or interviews completed with or by clinical professionals (doctor/pharmacist). Questions included hypothetical clinical situations, for example, *“would you prescribe an antibiotic to a patient with X symptoms”*. The responses were then compared with clinical prescribing guidelines and classified as appropriate or inappropriate use (proportion was calculated as inappropriate use over total responses). Doctors may also have been asked about their prescribing practices, and pharmacists asked whether they would dispense antibiotics to a patient in the absence of a prescription.

#### Behavioural studies/direct observations

These studies measured provider-mediated use of antibiotics and fell in two broad categories. In the first category, actors presented themselves as patients to pharmacies, doctors or informal healthcare workers (HCWs) with a specific set of symptoms, but lacked an antibiotic prescription and assessed whether the pharmacist dispensed them with antibiotics. In the second category, individuals shadowed pharmacists and recorded if antibiotics were given without a prescription, at the correct dosage/duration, etc. For this, all antibiotics obtained in the absence of a prescription (‘non-prescription’) were counted as inappropriate, irrespective of legal need for a prescription to obtain antibiotics in the country of the study.

Across the four study designs, indicators of inappropriate antibiotic use were grouped into seven categories. Five were related to provider-mediated use: (1) lack of indication, (2) incorrect drug selection, (3) incorrect treatment duration, (4) incorrect dosage and (5) incorrect route of administration. Two were related to patient use: (6) non-prescription from leftovers packages or sharing packages, self-prescription/self-medication with antibiotics (SMA) and included obtaining antibiotics from pharmacy without a prescription (where there was a legal need for one) and (7) non-compliance (eg, not completing a full course).

### Data analysis

Median inappropriate use prevalence was compared between national income level (as classified by World Bank in 2020), study designs, indicators and clinical specialties. We used beta regression to compare inappropriate use prevalence between groups due to the overdispersion in the response variable.^22^ Post hoc pairwise comparisons of prevalence were conducted using marginal contrasts, interpreted as the difference in proportions between the reference and comparator groups. All studies were assumed to be independent (ie, none were substudies within studies or had overlap of populations), and each was included as a separate unweighted data point in each of the analyses. As limited healthcare access was hypothesised to be a key explanator for differences in inappropriate use level between high-income countries (HICs) and LMICs, we included the number of physicians per 1000 population obtained from the World Bank,^23^ as a proxy for access to healthcare in the regression models and assessed whether it could explain the differences observed. Crude death rate per 1000 and hospital beds per 1000 (also obtained from World Bank) were also explored separately, as well as all three together in a multivariate beta regression model to assess whether they could explain the differences observed. Additional explanatory variables classified and obtained from the World Bank were included (gross domestic product per capita (in current US$), percentage of population using safe drinking water sources, percentage of urban population, poverty headcount at US$2.15 per day and life expectancy at birth), but these were not retained in the final model due to issues with collinearity (online supplemental file 1). All analyses were conducted using R V.4.1.2, using packages ‘betareg’ and ‘modelbased’.

### Global estimates of inappropriately used antibiotics

We used IQVIA’s MIDAS database on sales of antibiotics by country from 2022^9^ to estimate the global quantity of antibiotics potentially used inappropriately under three scenarios. The MIDAS dataset was chosen as it was the only publicly available global dataset, known to the authors, which contained worldwide estimates on the sales of antibiotics by country.

MIDAS collects monthly sales data from a sample of hospitals, clinics and retail outlets such as pharmacies from which the aggregate and per capita sales of antibiotics can be estimated for each country. As the MIDAS data is available for over 90 countries of the world, the analysis required extrapolation of estimates for the remaining countries with missing data to allow for entire global estimation. Several studies have previously used IQVIA MIDAS data to estimate the global sales of antibiotics.^9 24^

The proportion of antibiotics potentially used inappropriately globally was calculated by dividing the estimated inappropriate use of antibiotics in each country, as determined in each scenario, by the total antibiotic consumption for each country (in MIDAS), and then aggregating the results at the global level. CIs were calculated using a non-parametric bootstrap approach with 10 000 resamples, and the 95% CI was derived from the 2.5th and 97.5th percentiles of the bootstrap distribution.

First, we used the prevalence estimated from clinical audits to calculate a ‘conservative’ estimate of inappropriately used antibiotics worldwide. The motivation was twofold: clinical audits were considered the most robust data source because of their larger sample (n=208) compared with other methods (patient interviews (n=150), behavioural studies (n=24) and provider interviews (n=30)). Furthermore, clinical audits do not rely on reports from humans (ie, patient, doctor or nurse) which could be susceptible to recall bias or the influence of an interviewer such as through leading questions. Instead, clinical audits consist of comparisons between written records (actual prescription vs clinical prescribing guidelines) and represent provider-mediated use of antibiotics.

For the second scenario, we used the average prevalence of inappropriate use by country calculated from patient interviews, as this was the only study design to focus on patient use of antibiotics. For the third scenario, we used the average prevalence of inappropriate use by country calculated from studies using ‘lack of indication’ (unnecessary prescription) to categorise inappropriate use that was exclusively provider-mediated; this was hypothesised to be a key factor for driving inappropriate use. For the first two scenarios, for countries without data, we used an average prevalence calculated from countries with data in the same national income level (ie, LMICs or HICs) and corresponding study design. For the third scenario, this was calculated from all countries with data in the same national income level from across all study designs.

### Patient and public involvement

It was not relevant to include patients or the public in the design, or conduct, or reporting, or dissemination plans of our research.

## Results

**Figure 2:**
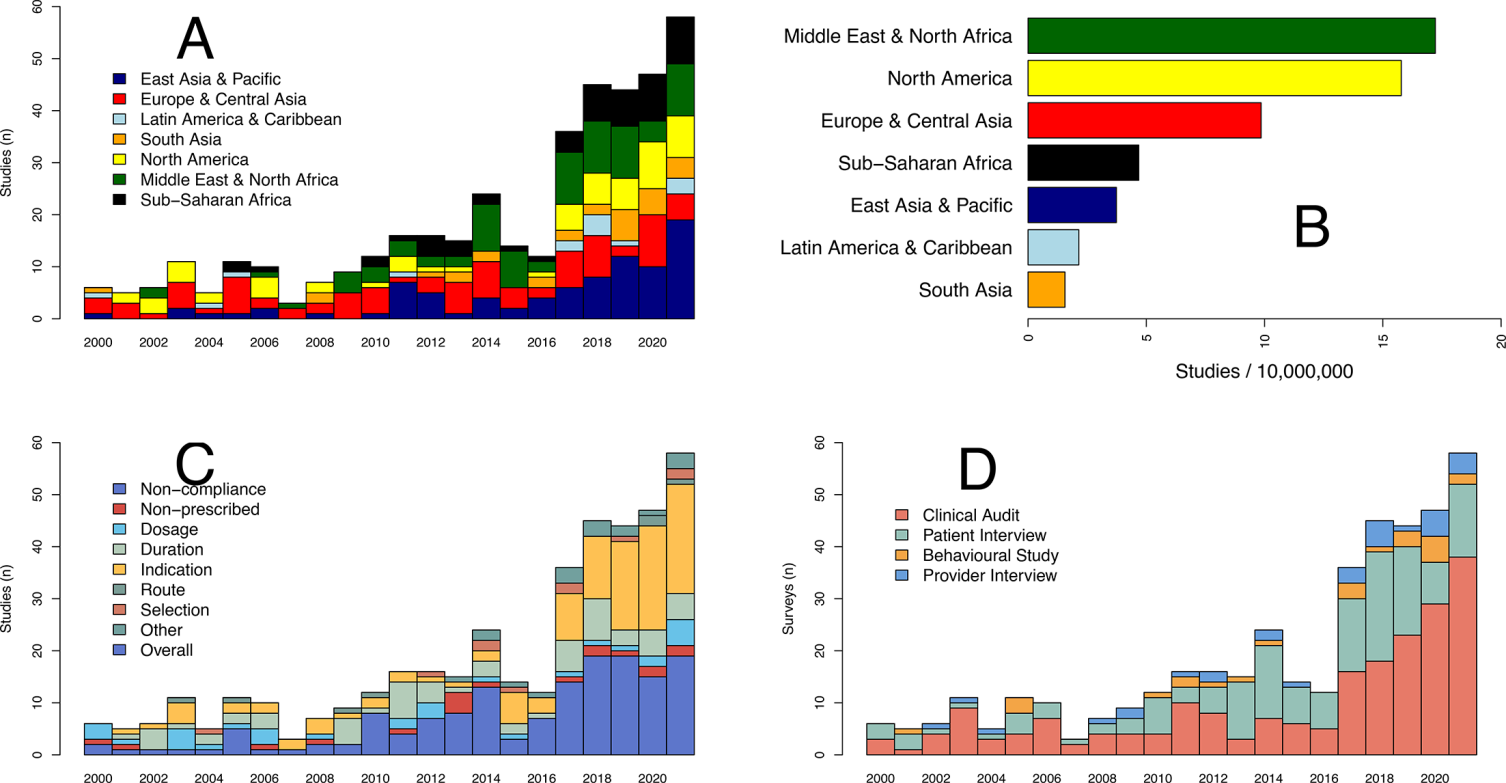
(**A**) Number of studies on inappropriate antibiotic use between 2000 and 2021, (**B**) number of studies per capita per world region between 2000 and 2021, (**C**) number of studies by indicator between 2000 and 2021, (**D**) number of studies by study design between 2000 and 2021.

Between 2000 and 2021, the number of studies on inappropriate antibiotic use increased from 6 to 58 per year (figure 2A). Of the 412 studies included, 200 studies were from HICs and 212 studies were from LMICs. We extracted 1033 estimates of inappropriate antibiotic use prevalence from studies conducted in 93 countries, including 299 estimates from Europe and Central Asia (28.9%), 225 from East Asia and Pacific (21.8%), 184 from Middle East and North Africa (17.8%), 137 from North America (13.3%), 105 from sub-Saharan Africa (10.2%), 50 from South Asia (4.8%) and 35 from Latin America (3.4%) (online supplemental figure 1). Per capita, the Middle East and North Africa and North America were the regions with the highest number of studies with >15 per 10 000 000 inhabitants, whereas Latin America, and East and South Asia had <4 per 10 000 000 inhabitants (figure 2B). The most frequent keywords used to describe the type of antibiotic use were inappropriate use (50.0%, 515/1033), followed by SMAs (11.0%, 114/1033). The keyword inappropriate was the most frequent wording, in all regions except South Asia, where irrational use dominated (34.0%, 17/50).

Provider-mediated indicators were used in 44.3% of estimates of inappropriate use, followed by 28% for patient use. The remaining 27.7% of estimates included mixed or unspecified indicators (figure 2C). For study designs, inappropriate antibiotic use was most frequently measured via clinical audits (n=208), and patient interviews (n=150), and less frequently via provider interview (n=30) and behavioural studies (n=24) (figure 2D).

**Figure 3:**
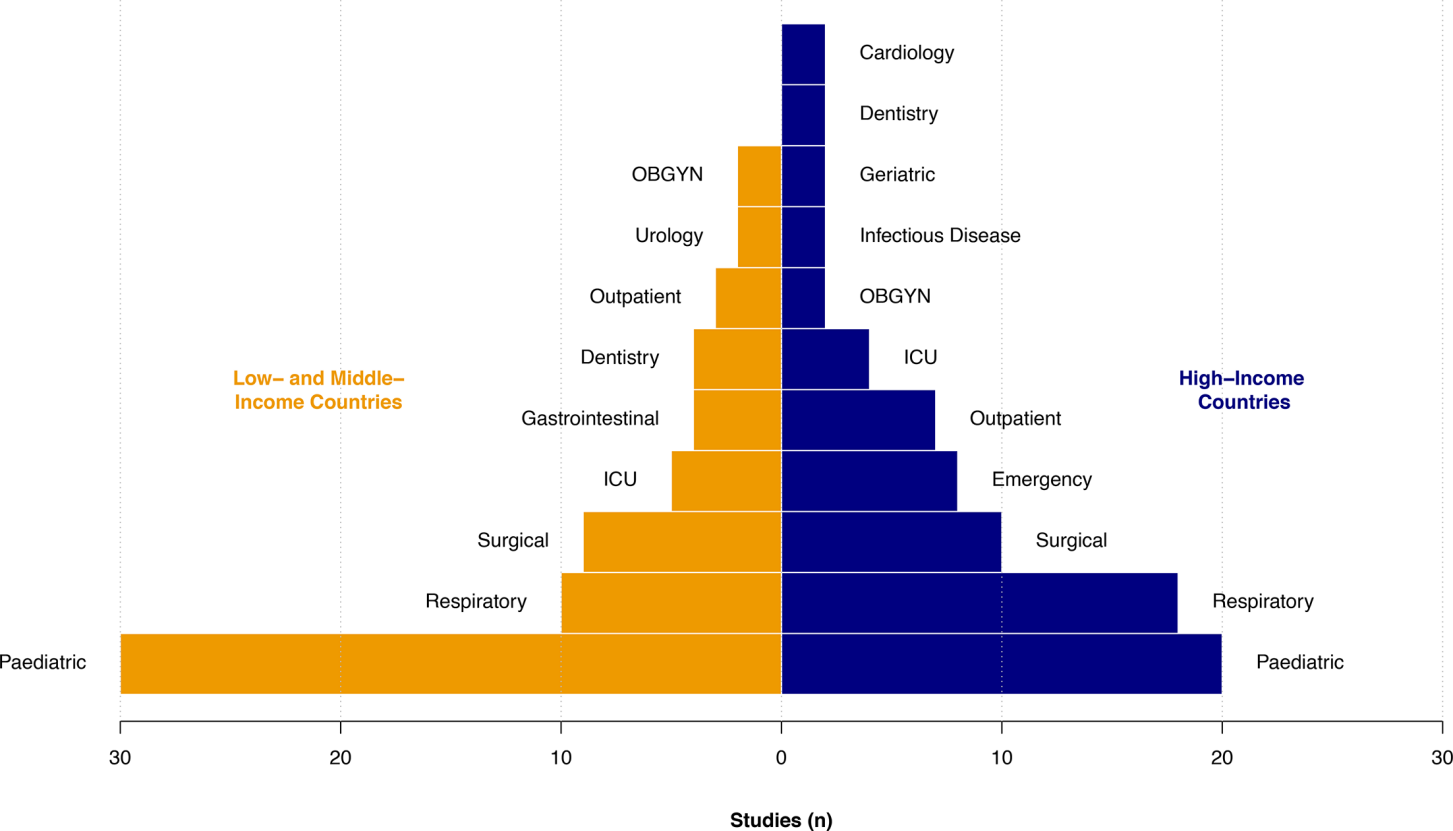
Number of studies on antibiotic misuse by clinical specialty (with at least two studies) and national income level. ICU, intensive care unit; OBGYN, obstetrics/gynaecology.

We identified studies conducted in the community, pharmacies, as well as outpatient and inpatient hospital settings. The majority (64%) of studies did not report clinical specialties. For those that did report a clinical specialty, the number of studies varied considerably by group (from n=126 in paediatrics and n=71 in respiratory, to only one in both gynaecology and neurology). The three most frequently reported clinical specialities were identical in HICs and LMICs: paediatrics, surgery and respiratory medicine (figure 3).

**Figure 4:**
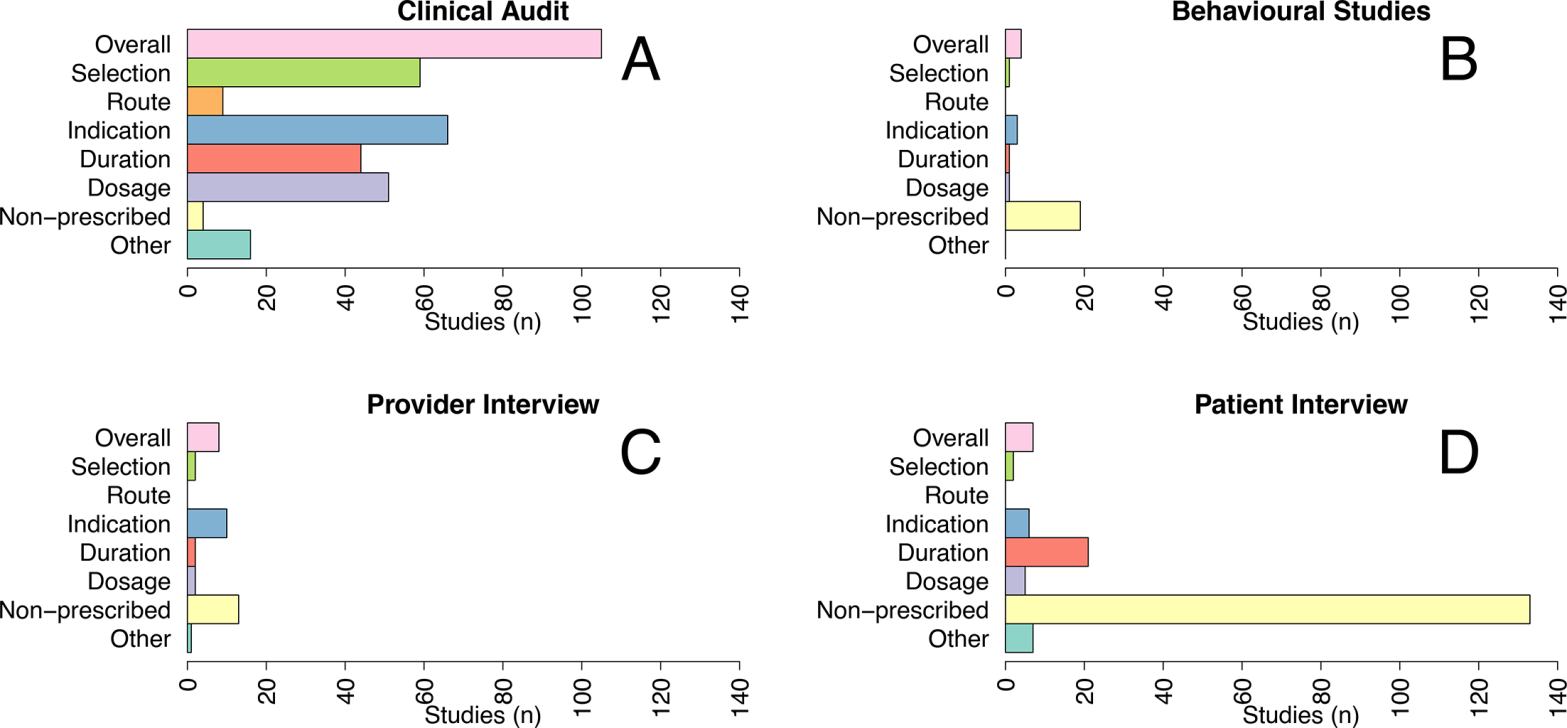
Number of studies on inappropriate antibiotic use by indicators and study design.

The indicators used for investigating prevalence varied by study design. For clinical audits, most commonly used was a combination of indicators followed by ‘lack of indication’ according to prescription guidelines (figure 4). For patient interviews, the most common indicator was the frequency of antibiotic use without prescription (‘non-prescribed’).

**Figure 5:**
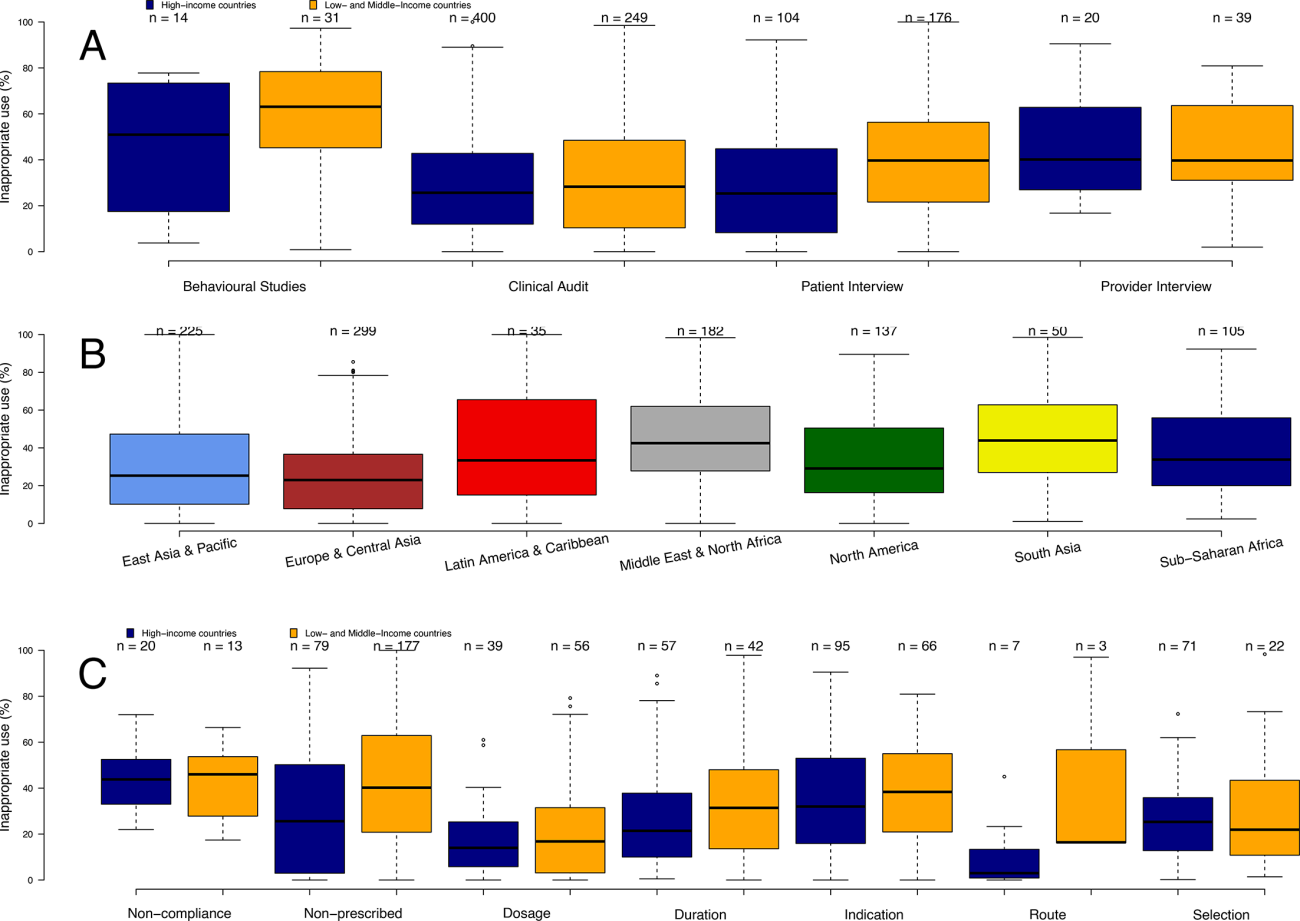
Inappropriate antibiotic use prevalence by (**A**) high-income and low-income and middle-income countries and study design, (**B**) by world region and (**C**) high-income and low-income and middle-income countries and indicator.

Median inappropriate use of antibiotics prevalence pooled across all study designs ranged from 23.0% (IQR: 7.8%–36.6%) in Europe to 43.9% (IQR: 27.3%–62.1%) in South Asia and 42.5% (IQR: 27.8%–61.6%) in Middle East and North Africa. However, all regions had large CIs which overlapped (figure 5). There was a statistically significant association between national income level (LMICs vs HICs) and prevalence of inappropriate antibiotic use (p<0.001). The post hoc analysis of marginal contrasts showed pairwise contrasts of inappropriate use prevalence between LMICs and HICs of 0.06 (95% CI 0.03, 0.09), indicating that LMICs had on average a 6% higher mean inappropriate antibiotic use prevalence than HICs. However, after controlling for the number of physicians per 1000 per country, this difference was no longer significant with a pairwise contrast of −0.01 (SE=0.02, 95% CI −0.06, 0.03) (online supplemental table 1).

Patient interviews were the only study design that had a statistically significant difference of inappropriate antibiotic use between LMICs and HICs (p<0.001, online supplemental table 1, figure 5A). Pairwise contrasts for LMICs were higher than HICs at 0.14 (SE=0.02, 95% CI 0.09, 0.20), indicating a 14% higher mean inappropriate use prevalence when estimated using data obtained from patient interviews only. After controlling for the number of physicians per 1000 per country, this difference was also no longer significant, with a pairwise contrast of 0.04 (SE=0.04, 95% CI −0.04, 0.12) (online supplemental table 1). The ‘non-prescription’ group was the only indicator with a significant difference between LMICs and HICs, where the post hoc pairwise contrast was 0.15 (95% CI 0.09, 0.21), indicating that LMICs had on average 15% higher mean prevalence of non-prescribed antibiotics than HICs.

Based on average prevalence obtained from clinical audits and patient interviews, we estimated that 29.5% (95% CI 25.1%, 35.4%) and 36.5% (95% CI 22.4%, 42.6%) of antibiotics consumed worldwide may be used inappropriately, respectively. We estimated that unnecessary prescription (‘lack of indication’) by HCWs accounted for 30.8% (95% CI 22.5%, 36.8%) of antibiotics consumed worldwide. In absolute terms, an average of ~30% of the global consumption of antibiotics would represent ~13 000 000 kg of active ingredient inappropriately used each year, or the equivalent of the annual antibiotic consumption of China.

## Discussion

Antibiotic consumption, a key driver of AMR, has received increasing attention, with several international surveillance initiatives launched to monitor its trends, such as the Global Antimicrobial Resistance Surveillance System (GLASS) and European Surveillance of Antimicrobial Consumption Network, as well as high-resolution mapping efforts.^25^ However, thus far, the inappropriate use of antibiotics has received comparatively less attention on a global scale. Here, we examined 412 studies reporting on inappropriate antibiotic use and attempted to estimate the quantity of antibiotic potentially used inappropriately globally each year.

The increase in the number of studies on inappropriate use between 2000 and 2021 highlights a growing attention to the issue in many parts of the world. We found the highest number of studies per region in Middle East and North Africa (n=182), with more than 52 from Saudi Arabia alone. Conversely, a recent study by Sweileh,^19^ which summarised bibliometric data of 656 published research and review articles and conference papers on ‘irrational’ use of antimicrobials, in any language, between 1980 and 2020, found the highest number of studies (n=140, 21.6%) were conducted in the USA. This difference with our findings could be attributable to their less specific search strategy, limited exclusion criteria or larger time frame. However, in line with our results, they also found the highest number of studies by clinical specialty in paediatrics and respiratory. Few studies were found in Western and subequatorial Africa and South America. One potential explanation for low numbers may be that our literature search was conducted in English. However, for a similar exercise synthesising literature on AMR in animals^26^—which included studies in English, Spanish, French and Portuguese—these same regions also showed comparatively lower numbers of studies than carried out in other regions.

Prevalence of inappropriate use was heterogeneous by study designs and indicators, and therefore, we estimated prevalence separately for specific study designs and indicators as well as pooled together. When pooled across all study designs, we estimated a ~6% higher prevalence of inappropriate use of antibiotics in LMICs compared with HICs, whereas when focusing specifically on patient interviews, this difference was ~14%. However, these differences disappeared after accounting for the number of physicians per capita to reflect access to healthcare/antibiotic prescriptions. In addition, we found that the ‘non-prescribed’ group, that is, those obtaining antibiotics without a prescription, was the only indicator where there was a significant difference observed between HICs and LMICs. Therefore, differences in inappropriate antibiotic use between HICs and LMICs were likely largely driven by use of antibiotics in the absence of a prescription. This highlights the considerable inequalities in access to health professionals between HICs and LMICs. Perhaps more importantly, it may reflect patients with appropriate indications for antibiotic use but lacking access to providers for prescriptions and does not consider the many informal healthcare providers and pharmacists who may have appropriately provided medication based on patient symptomatology despite a lack of prescription. These differences may also be driven by non-prescription use through a lack of or incomplete enforcement of regulations, inadequate knowledge by patient and/or provider, lack/underuse of diagnostic services, limited availability of antibiotics and/or lack of alternative treatments.^27 28^ However, as large differences still remain between and within countries, there are likely many other factors driving inappropriate use that cannot be accounted for within the scope of this study.

The multiplicity of study designs and indicators used to assess inappropriate use of antibiotics was a challenge for synthesising the studies identified in this scoping review. Kardas *et al*^11^ also identified this limitation in their systematic review—on antibiotic ‘misuse’ in the community—that data *‘varied in compliance or leftover definition, measurement technique, scientific rigour and study population’.* For patient interviews, we found a large number of studies reporting KAPs, that focused on different target groups that were not completely comparable: either clinical patients or the general community, and this may explain the wide range in estimates obtained from these studies in particular. Additionally, as questions were not standardised across surveys, responses could have been susceptible to interviewer influence, and therefore, results derived from these studies should be interpreted with caution. Therefore, we used data in three separate scenarios (clinical audits, patient interviews and ‘lack of indication’ indicator) to estimate a range on the global amount of antibiotics that is potentially used inappropriately.

We observed that a third of the antibiotics consumed globally are potentially due to be unnecessary prescriptions (‘lack of indication’) by HCWs, indicating the importance of antibiotic stewardship interventions. However, it cannot be ruled out that in a subset of these cases, there may have been inadequate reporting of the indication rather than a true lack of indication.^29^ For the other two scenarios, we identified that estimates were also around 30% of antibiotics consumed globally—whether used with the wrong indication, selection, dosage, or duration, or in the absence of a prescription. The most comparable of our global estimates (36.5%)—obtained from patient interviews (as the majority of indicators were ‘non-prescription’)—is in line with findings from previous systematic reviews, which included a larger number of studies, that found the prevalence of household antimicrobial self-medication to be 39% (95% CI 30%, 48%) in developing countries^30^ and lower than the use of non-prescribed antibiotics in LMICs to be 78% (95% CI 65%, 89%).^14^

There were several limitations in our study to consider. First, the diversity of indicators and study designs limited the scope for our modelling estimates. To further improve the robustness of information on the inappropriate use of antibiotics worldwide, there is an urgent need to define standardised definitions and indicators for classifying types of inappropriate use. This could be integrated into international initiatives, such as GLASS, and could include a standardised and validated survey instrument for collecting such data. For instance, defining a study design protocol akin to laboratory guidelines used for antimicrobial susceptibility testing under standard and reproducible protocols,^31^ which considers the specific population under study and relevant indicators to ensure comparability of study results. Notably, point prevalence surveys are currently the best alternative available in the absence of data obtained through systematic surveillance. Additionally, a hierarchical definition of the types of inappropriate use could be considered, based on the differing consequences, issues and potential interventions associated with the different types of inappropriate use.

Second, all studies we identified were cross-sectional, highlighting the potential for the development of a summary indicator that aggregates inappropriate use levels across drug classes, which can then be used to assess long-term trends and monitor progress in reducing inappropriate use.

Third, as studies were obtained through passive surveillance, the data may be subject to sampling bias as there may have been preferential sampling of inappropriate use behaviour of patients and physicians in certain regions (eg, Middle East), countries or clinical specialties. Regional variations in clinical guidelines and regulations may also introduce heterogeneity in what falls under the definition of inappropriate use. Fourth, national income-level status was assigned based on World Bank designation in 2020 and did not take into account countries transitioning between national income levels during the time period. Fifth, our ability to adjust for other potential factors that may influence inappropriate antibiotic use was limited by the availability of data. In particular, approaches such as task shifting to compensate for lack of human capital may not be captured by existing covariates. Finally, only a few studies reported drug classes studied, and therefore, it was not possible to describe studies by AWaRe classification of antibiotics.

Our findings have important policy implications. Although there are established clinical guidelines for appropriate antibiotic prescribing and sales—implemented by agencies such as national medical boards and health ministries—their availability, contents and enforcement vary across countries. Following WHO’s Global Action Plan for tackling AMR in 2015, some 148 countries have developed similar National Action Plans (NAPs) by 2021. However, a recent study by Chua *et al*^32^ found that NAPs suffered from weak design and implementation in many countries in Africa, South America and Asia. Among 55 countries in the African Union, only 20 had antibiotic treatment guidelines in 2021 but none of the guidelines adhered to the commonly used Grading of Recommendations Assessment, Development and Evaluation criteria for designing recommendations in healthcare.^33^ Recent efforts such as the WHO’s AWaRe Antibiotic Book of 2022^34^ have provided more detailed guidelines, listing diagnostic procedures and antibiotic use for common infections that are treated at primary care and hospital settings. NAPs and other national guidelines should adopt these recommendations.

Furthermore, future efforts for curbing inappropriate use of antibiotics should focus on raising awareness about AMR and enforcing antibiotics prescribing and dispensing regulations. This is especially important for LMICs where weak regulations and inadequate access to healthcare often lead to inappropriate prescribing by providers, without-prescription sales of antibiotics by pharmacies, and self-medication by consumers.^35^,^37^ Strengthening primary healthcare systems and reducing the burden of infectious diseases through improvements in water and sanitation access and routine vaccination coverage can also help reduce antibiotic use, including inappropriate use, substantially.

## Conclusions

To our knowledge, this study is the first to quantify the burden of inappropriate antibiotic use globally: ~13 000 000 kg in 2022, or ~30% of antibiotics used worldwide each year. The proportion of inappropriate antibiotic use was higher in LMICs than HICs. However, this could be largely attributable to differences in access to care, emphasising the need to account for limited access to healthcare providers in future research and policy endeavours. We observed that the attention given to, and prevalence of, inappropriate use of antibiotics was heterogeneous across regions and that a diversity of indicators and study designs was being used to quantify inappropriate use of antibiotics. Therefore, while further studies aiming to quantify inappropriate use are urgently needed, particularly in South America and South Asia, a necessary first step would be to define a set of standardised indicators, for both provider-mediated and patient use. Robust indicators would allow for tracking of progress in reducing inappropriate use of antibiotics and support further research on the drivers of inappropriate use and its role in driving AMR. These indicators could guide public health efforts to reduce AMU, allowing for the setting of realistic targets, particularly in LMICs,^38^ and in turn in reducing AMR worldwide.

## Supplementary material

10.1136/bmjph-2024-002411 online supplemental file 1

10.1136/bmjph-2024-002411 online supplemental file 2

## Data Availability

Data are available on reasonable request. All data relevant to the study are included in the article or uploaded as supplementary information.

## References

[1] Karakonstantis S, Kalemaki D (2019). Antimicrobial overuse and misuse in the community in Greece and link to antimicrobial resistance using methicillin-resistant S. aureus as an example. J Infect Public Health.

[2] Vibet M-A, Roux J, Montassier E (2015). Systematic analysis of the relationship between antibiotic use and extended-spectrum beta-lactamase resistance in Enterobacteriaceae in a French hospital: a time series analysis. Eur J Clin Microbiol Infect Dis.

[3] Goossens H, Ferech M, Vander Stichele R (2005). Outpatient antibiotic use in Europe and association with resistance: a cross-national database study. Lancet.

[4] Duong QA, Pittet LF, Curtis N (2022). Antibiotic exposure and adverse long-term health outcomes in children: A systematic review and meta-analysis. J Infect.

[5] Walsh TR, Gales AC, Laxminarayan R (2023). Antimicrobial Resistance: Addressing a Global Threat to Humanity. PLoS Med.

[6] Bao L, Peng R, Wang Y (2015). Significant reduction of antibiotic consumption and patients’ costs after an action plan in China, 2010-2014. PLoS One.

[7] Llor C, Bjerrum L (2014). Antimicrobial resistance: risk associated with antibiotic overuse and initiatives to reduce the problem. Ther Adv Drug Saf.

[8] Antimicrobial Resistance Collaborators (2022). Global burden of bacterial antimicrobial resistance in 2019: a systematic analysis. Lancet.

[9] Klein EY, Impalli I, Poleon S (2024). Global trends in antibiotic consumption during 2016-2023 and future projections through 2030. Proc Natl Acad Sci U S A.

[10] World Health Organization Promoting rational use of medicines. https://www.who.int/activities/promoting-rational-use-of-medicines.

[11] Kardas P, Devine S, Golembesky A (2005). A systematic review and meta-analysis of misuse of antibiotic therapies in the community. Int J Antimicrob Agents.

[12] Bortone B, Jackson C, Hsia Y (2021). High global consumption of potentially inappropriate fixed dose combination antibiotics: Analysis of data from 75 countries. PLoS One.

[13] Alhomoud F, Aljamea Z, Almahasnah R (2017). Self-medication and self-prescription with antibiotics in the Middle East-do they really happen? A systematic review of the prevalence, possible reasons, and outcomes. Int J Infect Dis.

[14] Torres NF, Chibi B, Kuupiel D (2021). The use of non-prescribed antibiotics; prevalence estimates in low-and-middle-income countries. A systematic review and meta-analysis. Arch Public Health.

[15] Torres NF, Chibi B, Middleton LE (2019). Evidence of factors influencing self-medication with antibiotics in low and middle-income countries: a systematic scoping review. Public Health (Fairfax).

[16] Pan H, Cui B, Zhang D (2012). Prior knowledge, older age, and higher allowance are risk factors for self-medication with antibiotics among university students in southern China. PLoS One.

[17] Mallah N, Orsini N, Figueiras A (2022). Income level and antibiotic misuse: a systematic review and dose-response meta-analysis. Eur J Health Econ.

[18] Morgan DJ, Okeke IN, Laxminarayan R (2011). Non-prescription antimicrobial use worldwide: a systematic review. Lancet Infect Dis.

[19] Sweileh WM (2021). Global research publications on irrational use of antimicrobials: call for more research to contain antimicrobial resistance. Global Health.

[20] Blaser MJ, Melby MK, Lock M (2021). Accounting for variation in and overuse of antibiotics among humans. Bioessays.

[21] Chan OSK, Lam W, Zhao S (2024). Why prescribe antibiotics? A systematic review of knowledge, tension, and motivation among clinicians in low-, middle- and high-income countries. Soc Sci Med.

[22] Ferrari S, Cribari-Neto F (2004). Beta regression for modelling rates and proportions. J Appl Stat.

[23] World Bank Physicians (per 1,000 people). https://data.worldbank.org/indicator/sh.med.phys.zs.

[24] Van Boeckel TP, Gandra S, Ashok A (2014). Global antibiotic consumption 2000 to 2010: an analysis of national pharmaceutical sales data. Lancet Infect Dis.

[25] Browne AJ, Chipeta MG, Haines-Woodhouse G (2021). Global antibiotic consumption and usage in humans, 2000-18: a spatial modelling study. Lancet Planet Health.

[26] Van Boeckel TP, Pires J, Silvester R (2019). Global trends in antimicrobial resistance in animals in low- and middle-income countries. Science.

[27] Otaigbe II, Elikwu CJ (2023). Drivers of inappropriate antibiotic use in low- and middle-income countries. JAC Antimicrob Resist.

[28] Sulis G, Sayood S, Gandra S (2022). Antimicrobial resistance in low- and middle-income countries: current status and future directions. Expert Rev Anti Infect Ther.

[29] Ladines-Lim JB, Fischer MA, Linder JA (2024). Prevalence of Inappropriate Antibiotic Prescribing with or without a Plausible Antibiotic Indication among Safety-Net and Non-Safety Net Populations. J Gen Intern Med.

[30] Ocan M, Obuku EA, Bwanga F (2015). Household antimicrobial self-medication: a systematic review and meta-analysis of the burden, risk factors and outcomes in developing countries. BMC Public Health.

[31] European Centre for Disease Prevention and Control (2016). EU protocol for harmonised monitoring of antimicrobial resistance in human Salmonella and Campylobacter isolates. https://www.ecdc.europa.eu/en/publications-data/eu-protocol-harmonised-monitoring-antimicrobial-resistance-human-salmonella-and-0.

[32] Chua AQ, Verma M, Hsu LY (2021). An analysis of national action plans on antimicrobial resistance in Southeast Asia using a governance framework approach. *Lancet Reg Health West Pac*.

[33] Craig J, Hiban K, Frost I (2022). Comparison of national antimicrobial treatment guidelines, African Union. Bull World Health Organ.

[34] World Health Organization The WHO AWaRe (access, watch, reserve) antibiotic book. https://www.who.int/publications/i/item/9789240062382.

[35] Auta A, Hadi MA, Oga E (2019). Global access to antibiotics without prescription in community pharmacies: A systematic review and meta-analysis. J Infect.

[36] Charani E, Smith I, Skodvin B (2019). Investigating the cultural and contextual determinants of antimicrobial stewardship programmes across low-, middle- and high-income countries-A qualitative study. PLoS One.

[37] Koya SF, Ganesh S, Selvaraj S (2022). Consumption of systemic antibiotics in India in 2019. Lancet Reg Health Southeast Asia.

[38] D’Atri F, Arthur J, Blix HS (2019). Targets for the reduction of antibiotic use in humans in the Transatlantic Taskforce on Antimicrobial Resistance (TATFAR) partner countries. Euro Surveill.

